# Association between the serum albumin-to-creatinine ratio and 28-day all-cause mortality in sepsis: a retrospective cohort study

**DOI:** 10.3389/fmed.2025.1540647

**Published:** 2025-09-04

**Authors:** Kerui Wu, Yi Yu, Li Li, Junxuan Wu, Jian Xu, Bojun Zheng, Dingwei Deng, Jian Li

**Affiliations:** ^1^Guangdong Provincial Hospital of Chinese Medicine, The Second Affiliated Hospital of Guangzhou University of Chinese Medicine, Guangzhou, China; ^2^The Second Clinical College of Guangzhou University of Chinese Medicine, Guangzhou, China; ^3^Guangdong Provincial Key Laboratory of Research on Emergency in Traditional Chinese Medicine, Guangzhou, China

**Keywords:** sepsis, albumin (ALB), creatinine (Cr), mortality, prognosis

## Abstract

**Objective:**

This study aimed to investigate the association between the serum albumin-to-creatinine ratio (ACR) and the prognosis of sepsis.

**Methods:**

Extracted clinical data of sepsis patients from the MIMIC-IV (v2.2) database. Based on the optimal ACR cutoff value, we divided the cohort into two groups and preformed propensity score matching to balance baseline characteristics. Explored the relationship between ACR and 28-day all-cause mortality using Cox proportional risk regression and Kaplan–Meier survival curves, and conducted subgroup analysis to evaluate the effect modifications across different patient populations. Applied the restricted cubic spline (RCS) curves to assess the nonlinear relationships, and the receiver operating characteristic (ROC) curve to assess the predictive performance.

**Results:**

After screening and matching, a total of 1,418 sepsis patients were included. Cox regression and Kaplan–Meier analysis showed that a high ACR value might be associated with a low 28-day mortality risk. Subgroup analysis revealed a significant interaction between age and ACR, as well as renal disease and ACR. RCS analysis revealed a nonlinear association between ACR values and reduced mortality risk. When ACR was below 2,300, there was a negative association between ACR and mortality. However, no significant association was observed when ACR exceeded 2,300. ROC curve analysis indicated that combining ACR with age, sex, body mass index, SOFA score, white blood cell, hemoglobin, blood lactate improved the predictive performance for 28-day all-cause mortality (AUC = 0.730).

**Conclusion:**

A higher ACR value may associated with a lower 28-day all-cause mortality risk when ACR value was less than 2,300. Moreover, ACR had some predictive power for adverse outcomes in sepsis.

## 1 Introduction

Sepsis is a life-threatening organ dysfunction resulting from a dysregulated host response to infection (1). If left untreated in the early stage, the condition may progress to life-threatening complications such as septic shock and multiple organ dysfunction syndrome. Despite advances in modern medical technology, the incidence and mortality rates of sepsis remain high. According to the World Health Organization, sepsis claims millions of lives annually worldwide, emerging as a major global health threat (2).

Serum albumin and serum creatinine are routine clinical biochemical markers and play important roles in assessing disease severity and prognosis in sepsis patients. Serum albumin, a key plasma component, plays a vital role in maintaining blood volume, regulating osmotic pressure, and facilitating nutrient transport. Its levels reflect nutritional status and immune function, with hypoalbuminemia strongly associated with increased mortality in sepsis (3–5). However, serum albumin level can be affected by chronic inflammation and renal function. Hence, albumin level alone may be insufficient for accurate prognostic assessment (6, 7). Serum creatinine, an indicator of renal function, is related to oxidative stress, endothelial function and inflammatory response (8). Sepsis frequently leads to renal impairment, and elevated serum creatinine level correlating strongly with disease severity and poor prognosis (9). However, abnormal serum creatinine level may also result from chronic kidney disease. Similarly, creatinine level alone is inadequate for disease prediction (10).

To better account for the effect of renal function on albumin, researchers proposed the albumin-to-creatinine ratio (ACR) as a more reliable indicator than serum albumin alone (11). The combined detection of serum albumin and creatinine can evaluate the inflammation levels, nutritional status and renal function, so as to fully understand the condition of patients and overcome the limitation that a single index is easily affected by various factors (12). Prior studies revealed that lower ACR was associated with a higher mortality risk in patients with ST-segment elevation myocardial infarction, heart failure, and those undergoing carotid stenting (11, 13, 14).

Since sepsis patients often present with renal impairment, the serum albumin level and creatinine level tend to exhibit inverse associations with sepsis prognosis. In this study, ACR is hypothesized to have a stronger predictive ability for the prognosis of sepsis. Therefore, this study utilized the MIMIC-IV (v2.2) database to analyze hospitalized sepsis patients, evaluating the relationship between ACR and 28-day all-cause mortality.

## 2 Methods and materials

### 2.1 Data sources

Obtain the data from the MIMIC-IV (v2.2) database, a public, large-scale clinical database open to researchers worldwide. The database includes medical information, such as length of stay, vital signs, laboratory test results, medications and outcomes, of more than 200,000 patients admitted to Beth Israel Deaconess Medical Center in Boston, Massachusetts, from 2008 to 2019. All patient information was de-identified, and the Institutional Review Board determined that informed consent was not necessary. The author (YY) has been granted access to this database (certificate ID number 6477678).

### 2.2 Inclusion criteria

1. Age greater than 18 years.
2. Diagnosis of sepsis with International Statistical Classification of Diseases and Related Health Problems 9th edition diagnostic codes 99,591, 99,592, and 10th edition diagnostic codes R652, R650, R6521.
3. Admission to the ICU.

### 2.3 Exclusion criteria

1. ICU stay of less than 1 day.
2. For patients who were admitted to the ICU multiple times for sepsis, only the first hospitalization data were included.
3. Patients who were not tested for serum albumin and serum creatinine within 24 h of ICU admission.

### 2.4 Outcome indicators

The primary outcome indicator was 28-day all-cause mortality.

### 2.5 Data extraction

PostgreSQL software^1^ and Navicat Premium software^2^ were used for data extraction, which was obtained by running Structured Query Language. STATA software (version 16.0) was applied for data integration and processing. The primary study variable was the serum albumin-to-creatinine ratio (ACR), defined as the ratio of the initial serum albumin test value (g/dL) to the initial serum creatinine test value (mg/dL) within 24 h of ICU admission. The demographic information included age, gender, height, weight, body mass index (BMI), vital signs included heart rate (HR), respiratory rate (RR), mean arterial pressure (MAP), peripheral blood oxygen (SPO_2_), laboratory parameters included white blood cells (WBC), hemoglobin (HB), platelets (PLT), serum creatinine (CR), serum urea nitrogen (UN), serum glucose (GLU), anion gap (AG), calcium (Ca^2+^), sodium (Na^+^), chloride (Cl^−^), potassium (K^+^), lactate (LAC), prothrombin time (PT), activated partial thromboplastin time (APTT), glutamic pyruvic transaminase (ALT), glutamic oxaloacetic transaminase (AST), and total bilirubin (TBIL). Information on co-morbidities included heart failure, chronic pulmonary disease, liver disease, diabetes, renal disease, and malignancy. In addition, information on the use of vasoactive drugs was extracted, primarily including norepinephrine, vasopressin, epinephrine, phenylephrine, dopamine, and dobutamine. The Sequential Organ Failure (SOFA) score was extracted to assess disease severity. All laboratory indicators and scores originated from the first test or evaluation after admission.

### 2.6 Missing values and outlier value handling

Variables with more than 15% missing values were excluded. For variables with missing values <15%, multiple interpolation was used, and the best set of data was selected for filling. Variables with outliers were subjected to Winsorization, in which values above the 99th percentile and below the 1st percentile were replaced with the 99th percentile and 1st percentile values, respectively.

### 2.7 Grouping and propensity score matching

The optimal ACR cutoff value for determining whether death occurred on the 28th day after hospitalization was 1846.154. Based on this optimal cutoff, the study patient population was divided into two groups: the low ACR group (Q1, ACR ≤1846.154) and the high ACR group (Q2, ACR >1846.154). The optimal cutoff point, which was the boundary where the Youden’s index was maximized, as shown in Supplementary Figure 1 and Supplementary Table 1.

To reduce the impact of potential confounders, we employed propensity score matching. The propensity scores were calculated using logistic regression. We performed 1:1 greedy nearest neighbor matching with a caliper of 0.075. The method functionally relied on the R package MatchIt. Used the standardized mean difference (SMD) to evaluate the balance of baseline covariates before and after matching. An absolute SMD value of less than 0.1 indicated good balance.

### 2.8 Statistical analysis

Continuous variables following a normal distribution were reported as mean ± standard deviation, medians and quartiles for non-normal distributed continuous variables, and case counts (percentages) for categorical variables.

Pearson’s chi-squared test was used to compare the 28-day all-cause mortality, and Kaplan–Meier survival curves was plotted to compare the 28-day survival. Subsequently, to assess the effect of ACR on survival time, we preformed the COX proportional risk regression. The proportional hazard assumption of COX regression model was tested based on Schoenfeld residuals, when *p-*value >0.05 indicated that the proportional hazard assumption was satisfied. To evaluate the impact of patient demographics, disease severity and clinical test indicators on prognosis, a multivariate Cox regression model was adjusted for 7 covariates (Model 1: no covariates were adjusted; Model 2: adjusted for age, gender, and BMI; Model 3: adjusted for age, gender, BMI, and SOFA score; Model 4: adjusted for age, gender, BMI, SOFA score, WBC, HBG, and LAC). We primarily selected covariates based on clinical knowledge and previous research. We assessed multicollinearity among covariates using the variance inflation factor (VIF) and excluded variables with VIF >5.

We performed restricted cubic spline (RCS) model to examine the nonlinear relationship between ACR and survival rates. Knots ranging from 3 to 7 were tested, and the model with the lowest Akaike information criterion (AIC) value was selected for RCS analysis. To assess the overall association between ACR and mortality, we examined the global *p*-overall value. *p*-overall value <0.05 indicates a statistically significant overall association between ACR and mortality, suggesting that the relationship modeled by the RCS differs significantly from a null hypothesis of no association. Additionally, we assessed whether the association between ACR and mortality was nonlinear. The *p*-nonlinear value was evaluated, where *p*-nonlinear value <0.05 indicates a statistically significant nonlinear relationship. If there was a nonlinear relationship, the segmented model was applied using the R segmented package, which estimates breakpoints and fits a separate linear model for each segment defined by these breakpoints. The value of breakpoints was determined based on model fit and statistical tests. To assess the overall association between ACR and mortality, the standard linear regression model was compared to the piecewise linear regression model. The log-likelihood ratio test (LRT) was used to determine if the piecewise model provided a significantly better fit than the standard linear regression model, with *p-*value <0.05 indicating a statistically significant difference.

Subgroup analysis evaluated the consistency between ACR and mortality. These analysis were stratified by age, gender, SOFA score, liver disease, renal disease. The participants were divided into two age groups based on a cutoff of 65 years (<65 and ≥65). The results were visualized using a forest plot, presenting the hazard ratios (HR) and 95% confidence intervals (CI). Statistical evaluations were further performed to assess interactions between subgroups.

We preformed the receiver operating characteristic (ROC) curve analysis of the SOFA score and Models 1 to 4, and the area under the curve (AUC) was calculated. The SOFA score was the most commonly used indicator for predicting mortality in sepsis in clinical practice. By comparing the AUC to evaluate the predictive performance of Models 1 to 4 for 28-day all-cause mortality of sepsis. The DeLong test was used to evaluate differences between AUC values.

All statistical analyses were performed using the R software (version 4.2.2), along with the MSTATA software.^3^ The two-sided test suggested that *p-*value <0.05 was statistically significant.

## 3 Results

### 3.1 Data screening and grouping

**Figure 1:**
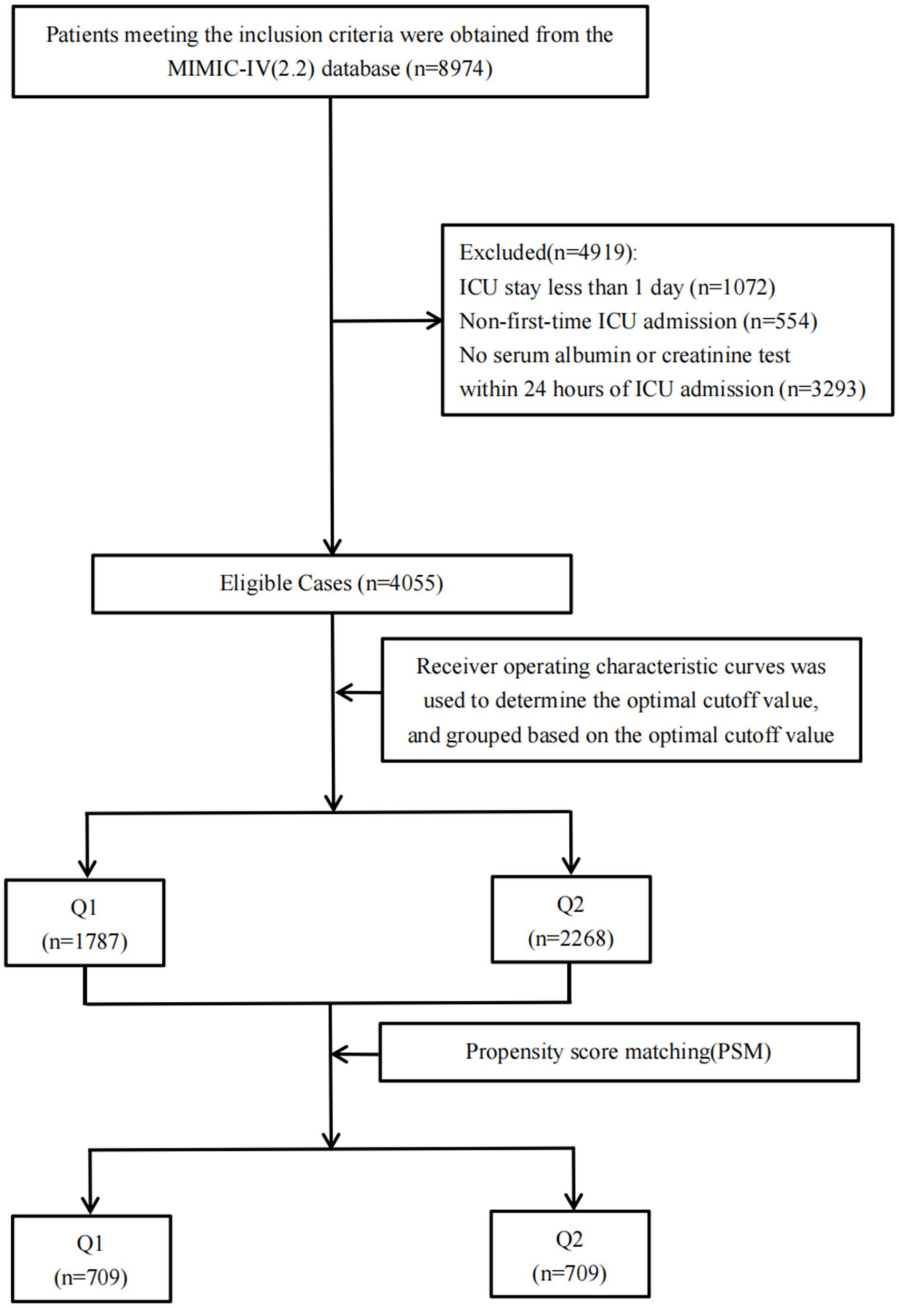
The data screening and grouping process.

We initially extracted 8,974 cases meeting the inclusion criteria from the MIMIC-IV (v2.2) database. After excluding 1,072 cases with ICU stays shorter than 1 day, 554 non-first-time ICU admissions, and 3,293 cases lacking serum albumin or creatinine tests within 24 h of admission, a total of 4,055 cases were ultimately included in the study. These cases were stratified into two groups based on the optimal ACR cutoff value. Following propensity score matching, 1,418 patients were successfully matched, with 709 patients in each group, as illustrated in Figure 1.

### 3.2 Baseline data

Table 1 presented the baseline characteristics of the study subjects after PSM. The standardized mean difference (SMD) for all baseline covariates was below 0.1, indicating well-balanced of baseline covariates between groups.

**Table 1:** Patient demographics and baseline characteristics.

Characteristics	Unmatched			Matched		
	Q1 *N* = 1,787	Q2 *N* = 2,268	SMD^a^	Q1 *N* = 709	Q2 *N* = 709	SMD^a^
Age						
Mean ± SD	68 ± 15	66 ± 17	0.095	68 ± 16	69 ± 15	−0.059
Gender (*n* %)						
Male	1,096 (61%)	1,162 (51%)	0.207	416 (59%)	407 (57%)	0.026
Female	691 (39%)	1,106 (49%)	−0.207	293 (41%)	302 (43%)	−0.026
Height (m)						
Mean ± SD	1.69 ± 0.11	1.67 ± 0.11	0.185	1.68 ± 0.11	1.68 ± 0.11	0.040
Weight (kg)						
Mean ± SD	86 ± 28	78 ± 24	0.291	82 ± 24	82 ± 26	0.010
BMI (kg/m^2^)						
Mean ± SD	30 ± 9	28 ± 8	0.241	29 ± 8	29 ± 8	−0.002
SOFA score						
Median (Q1, Q3)	10.0 (7.0, 14.0)	7.0 (4.0, 10.0)	0.738	9.0 (6.0, 12.0)	9.0 (6.0, 12.0)	0.006
Heart rate						
Mean ± SD	92 ± 18	92 ± 17	−0.019	92 ± 18	93 ± 18	−0.026
MBP (mmHg)						
Mean ± SD	72 ± 9	75 ± 10	−0.297	74 ± 10	74 ± 9	−0.003
Respiratory rate						
Mean ± SD	21.3 ± 4.6	21.1 ± 4.4	0.053	21.2 ± 4.4	21.2 ± 4.3	−0.016
SPO_2_ (%)						
Median (Q1, Q3)	97.00 (95.00, 98.00)	97.00 (95.00, 98.00)	−0.061	97.00 (95.00, 98.00)	97.00 (95.00, 98.00)	−0.030
Glucose (mmol/L)						
Median (Q1, Q3)	7.50 (6.00, 9.90)	7.20 (5.90, 9.00)	0.174	7.30 (6.00, 9.40)	7.60 (6.30, 9.60)	−0.019
WBC (10^9^/L)						
Median (Q1, Q3)	14 (9, 20)	12 (8, 18)	0.135	14 (9, 19)	13 (9, 18)	0.016
HGB (g/dL)						
Mean ± SD	9.87 ± 1.91	10.35 ± 2.03	−0.254	10.06 ± 2.00	10.08 ± 2.06	−0.009
PLT (10^9^/L)						
Median (Q1, Q3)	167 (101, 252)	185 (120, 270)	−0.149	173 (113, 254)	170 (103, 249)	0.020
Anion gap (mmol/L)						
Mean ± SD	18.7 ± 5.2	14.8 ± 3.3	0.757	16.5 ± 4.1	16.4 ± 3.5	0.022
UN (mg/dL)						
Median (Q1, Q3)	47 (34, 67)	21 (14, 29)	1.016	34 (25, 44)	32 (23, 44)	0.085
Calcium (mmol/L)						
Mean ± SD	8.01 ± 0.98	8.06 ± 0.76	−0.050	8.04 ± 1.04	8.08 ± 0.80	−0.043
Chloride (mmol/L)						
Mean ± SD	103 ± 8	104 ± 6	−0.159	104 ± 7	104 ± 7	−0.017
Sodium (mmol/L)						
Mean ± SD	137.6 ± 6.5	138.1 ± 5.4	−0.081	137.9 ± 5.8	137.9 ± 5.9	−0.011
Potassium (mmol/L)						
Mean ± SD	4.50 ± 0.77	4.11 ± 0.61	0.505	4.32 ± 0.75	4.30 ± 0.65	0.025
Lactate (mmol/L)						
Mean ± SD	3.17 ± 2.72	2.36 ± 1.78	0.299	2.81 ± 2.04	2.89 ± 2.17	−0.028
PT(s)						
Median (Q1, Q3)	17 (14, 23)	15 (13, 18)	0.267	16 (14, 21)	16 (14, 22)	−0.015
APTT(s)						
Median (Q1, Q3)	36 (31, 50)	33 (29, 40)	0.286	34 (30, 45)	35 (30, 45)	−0.013
ALT (U/L)						
Median (Q1, Q3)	32 (18, 92)	31 (17, 60)	0.194	30 (17, 62)	31 (18, 70)	−0.024
AST (U/L)						
Median (Q1, Q3)	56 (29, 156)	44 (26, 85)	0.192	48 (25, 109)	48 (27, 101)	−0.016
Total bilirubin (mg/dL)						
Median (Q1, Q3)	0.9 (0.5, 2.5)	0.8 (0.4, 1.7)	0.187	0.8 (0.5, 2.0)	0.9 (0.5, 2.3)	−0.003
Co-morbidity (*n* %)						
Congestive heart failure						
0	1,244 (70%)	1,693 (75%)	−0.109	510 (72%)	510 (72%)	0.000
1	543 (30%)	575 (25%)	0.109	199 (28%)	199 (28%)	0.000
Chronic pulmonary disease						
0	1,345 (75%)	1,671 (74%)	0.037	521 (73%)	516 (73%)	0.016
1	442 (25%)	597 (26%)	−0.037	188 (27%)	193 (27%)	−0.016
Liver disease						
0	1,308 (73%)	1,846 (81%)	−0.185	538 (76%)	538 (76%)	0.000
1	479 (27%)	422 (19%)	0.185	171 (24%)	171 (24%)	0.000
Diabetes						
0	1,097 (61%)	1,640 (72%)	−0.224	467 (66%)	467 (66%)	0.000
1	690 (39%)	628 (28%)	0.224	242 (34%)	242 (34%)	0.000
Renal disease						
0	1,036 (58%)	2,027 (89%)	−0.636	508 (72%)	510 (72%)	−0.006
1	751 (42%)	241 (11%)	0.636	201 (28%)	199 (28%)	0.006
Cancer						
0	278 (16%)	390 (17%)	0.045	125 (18%)	114 (16%)	−0.043
1	1,509 (84%)	1,878 (83%)	−0.045	584 (82%)	595 (84%)	0.043
Vasoactive drug (*n* %)						
0	1,143 (64%)	1,467 (65%)	−0.015	455 (64%)	446 (63%)	0.026
1	644 (36%)	801 (35%)	0.015	254 (36%)	263 (37%)	−0.026
Albumin (g/dL)						
Mean ± SD	2.74 ± 0.64	3.02 ± 0.63	0.441	2.66 ± 0.63	3.06 ± 0.65	0.625
Creatinine (mg/dL)						
Median (Q1, Q3)	2.50 (1.85, 3.70)	0.95 (0.70, 1.25)	1.386	1.95 (1.60, 2.70)	1.25 (1.00, 1.45)	0.899

BMI, body mass index; SOFA, sequential organ failure assessment; MAP, mean arterial pressure; SPO_2_, peripheral blood oxygen; WBC, white blood cell; HGB, hemoglobin; PLT, platelet; UN, serum urea nitrogen; PT, prothrombin time; APTT, activated partial thromboplastin time; ALT, alanine aminotransferase; AST, aspartate aminotransferase.

a Standardized mean difference.

### 3.3 Clinical outcome

**Figure 2:**
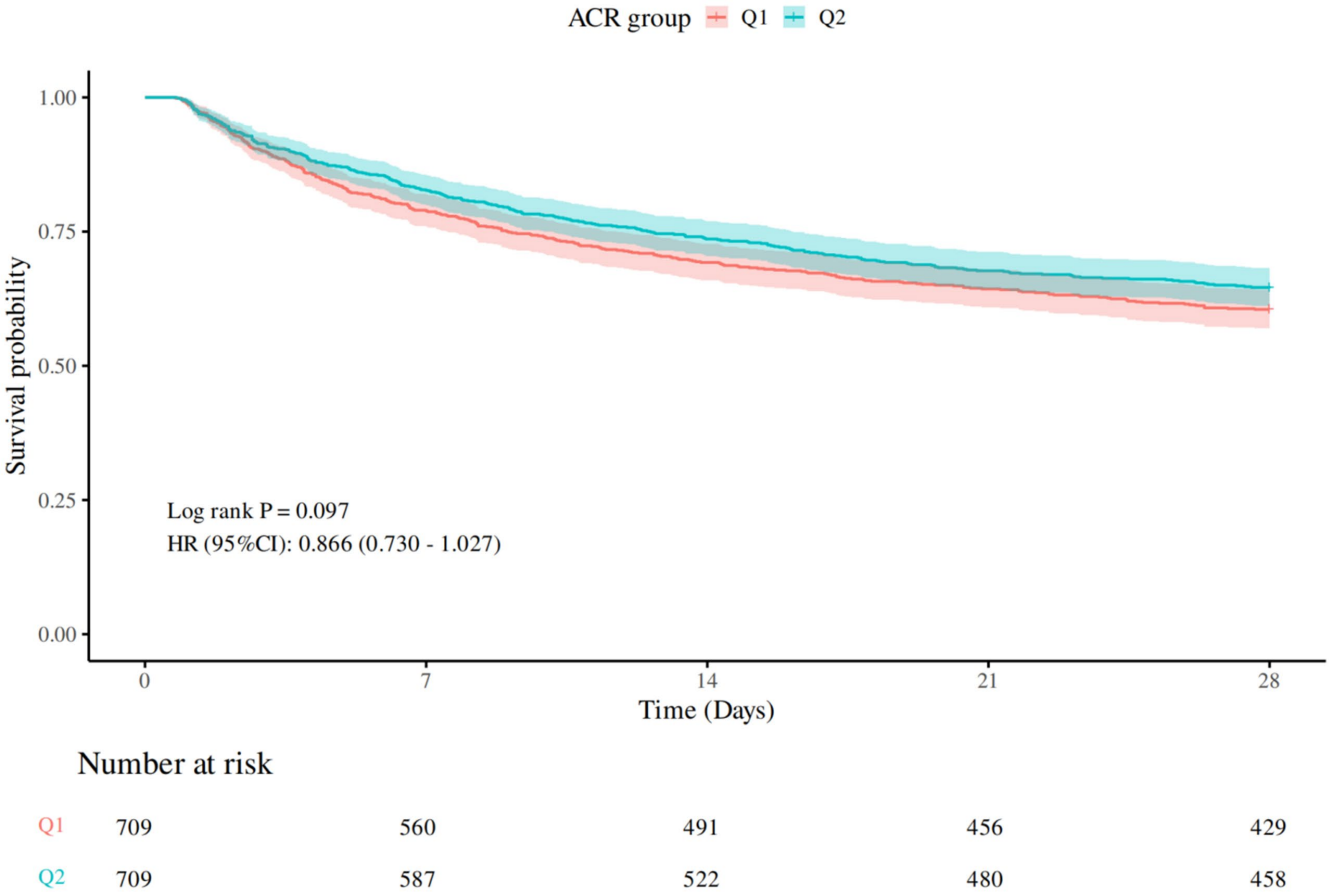
28-day Kaplan–Meier survival curve. Q1 (ACR ≤1846.154), Q2 (ACR>1846.154).

Table 2 presented the comparison of 28-day all-cause mortality between groups, and the Kaplan–Meier survival curve was depicted in Figure 2. Compared to Q1, there was a lower mortality rate in Q2. However, no statistically significant difference was observed (*p* > 0.05).

**Table 2:** 28-day all-cause mortality.

Outcome	Unmatched			Matched		
	Q1 *N* = 1,787	Q2 *N* = 2,268	*p*-value^a^	Q1 *N* = 709	Q2 *N* = 709	*p*-value^a^
Mortality (*n* %)			<0.001			0.125
0	986 (55%)	1,685 (74%)		429 (60.5%)	457 (64.5%)	
1	801 (45%)	583 (26%)		280 (39.5%)	252 (35.5%)	

a Pearson’s chi-squared test.

In the Cox regression analysis examining the association between ACR groups and mortality, participants in Q2 exhibited a lower hazard of mortality compared to the reference group (Q1) across all adjusted models. In the unadjusted model (Model 1), the hazard ratio (HR) for Q2 was 0.87 (95% CI: 0.73–1.03, *p* = 0.097). After adjusting for age, gender, and BMI (Model 2), the HR remained similar at 0.86 (95% CI: 0.72–1.02, *p* = 0.075). Further adjustment for SOFA score (Model 3) strengthened the association, yielding an HR of 0.82 (95% CI, 0.69–0.97, *p* = 0.021). Additional adjustments for laboratory markers (Model 4) maintained the significant protective effect, with HR of 0.81 (*p* = 0.016). These findings suggested that higher ACR level (Q2) was associated with a reduced risk of mortality, independent of clinical and demographic confounders (see Table 3).

**Table 3:** Association between ACR group and 28-day all-cause mortality (Cox regression).

Characteristic	Model 1			Model 2			Model 3			Model 4		
	HR	95% CI	*p*-value	HR	95% CI	*p*-value	HR	95% CI	*p*-value	HR	95% CI	*p*-value
ACR group												
Q1	—	—		—	—		—	—		—	—	
Q2	0.87	0.73, 1.03	0.097	0.86	0.72, 1.02	0.075	0.82	0.69, 0.97	0.021	0.81	0.68, 0.96	0.016

CI, confidence interval; HR, hazard ratio.

Model 1: no covariates were adjusted.

Model 2: adjusted for age, gender, and BMI.

Model 3: adjusted for age, gender, BMI, and SOFA score.

Model 4: adjusted for age, gender, BMI, SOFA score, white blood cell, hemoglobin, and lactate.

Proportional hazard assumption test revealed that the *p-*values for Model 1 to Model 4 were above 0.05, as detailed in Supplementary Table 2. This indicated that four models meet the proportional risk hypothesis and were suitable for COX regression analysis. All variance inflation factors (VIFs) of the covariates were less than 5, indicating no evidence of multicollinearity among the variables, as shown in Supplementary Table 3.

### 3.4 Restricted cubic spline analysis

**Figure 3:**
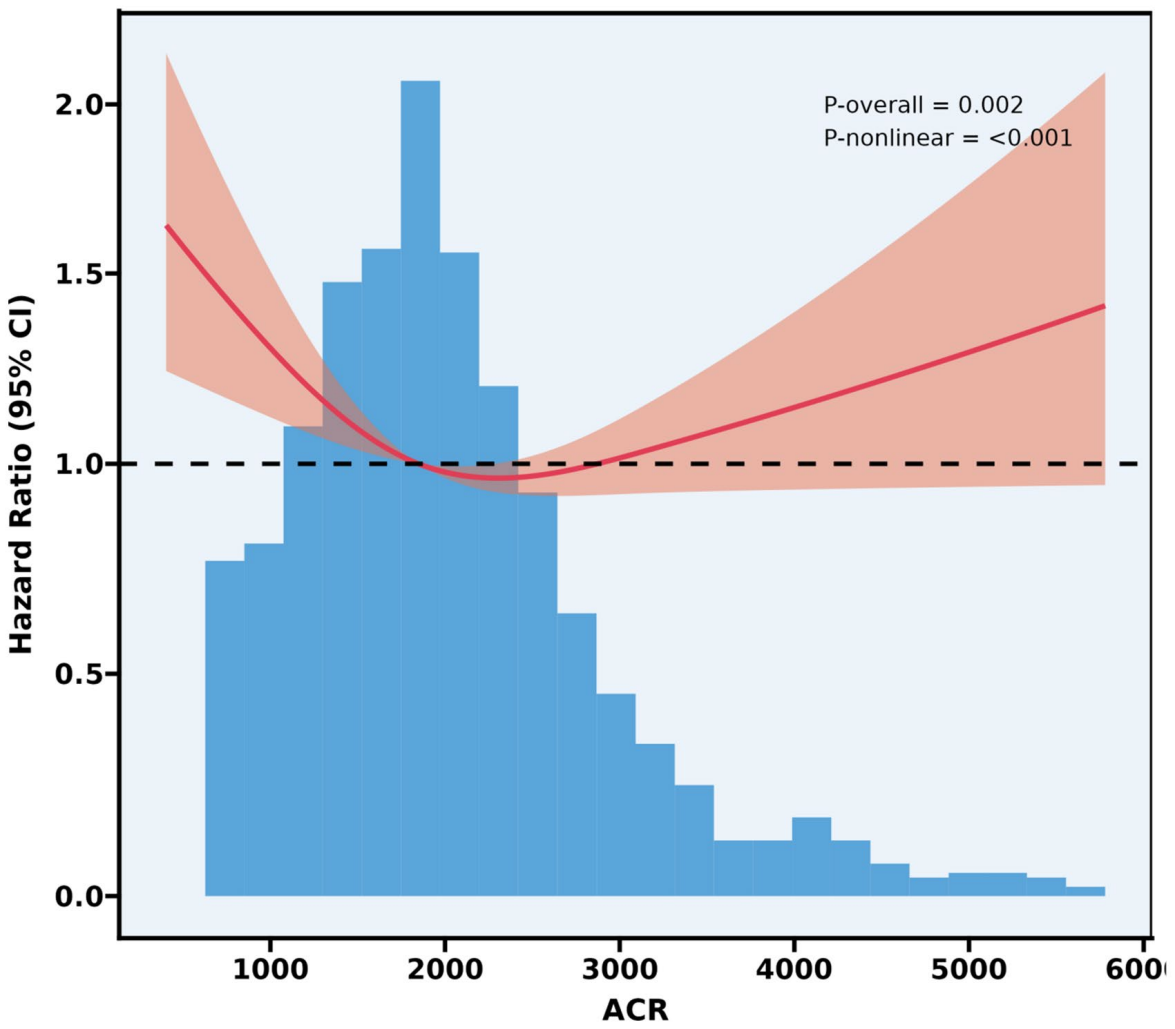
RCS curve for association between ACR and 28-day all-cause mortality. Model with 3 knots located at 10th, 50th and 90th percentiles. Y-axis represents the HR to present mortality for any value of ACR compared to individuals with reference value (50th percentile) of ACR. The Cox regression was adjusted for age, gender, BMI, SOFA score, white blood cell, hemoglobin, lactate.

The restrictive cubic spline curve illustrated the relationship between the ACR and the mortality hazard ratio, as shown in Figure 3. As ACR increased from a lower value, the curve initially exhibited a decreasing trend, reaching a minimum around an ACR of 2,000. Following this minimum point, the slope of the curve shifted, indicating an upward trend as ACR continues to increase. This analysis was statistically significant, with an overall *p*-value of 0.002, suggesting a meaningful relationship between ACR and the mortality hazard ratio. Moreover, the *p*-value for nonlinearity was less than 0.001, further supporting the presence of a nonlinear association.

Supplementary Table 4 revealed the results of piecewise Cox regression. The standard Cox regression analysis revealed a non-significant association between ACR and mortality (adjusted HR 1.0000, 95% CI 0.9999–1.0001, *p* = 0.378). However, piecewise Cox regression identified a significant break-point at ACR = 2,300 (*p* = 0.005 by likelihood ratio test). Below this threshold, each unit increase in ACR was associated with a significantly decreased mortality risk (adjusted HR 0.9997, 95% CI 0.9995–0.9999, *p* = 0.007). Conversely, above the break-point, no association between ACR and mortality was found (adjusted HR 1.0001, 95% CI 1.0000–1.0002, *p* = 0.130). The piecewise model demonstrated significantly better fit compared to the linear model (likelihood ratio test *p* = 0.005).

### 3.5 Subgroup analysis

**Figure 4:**
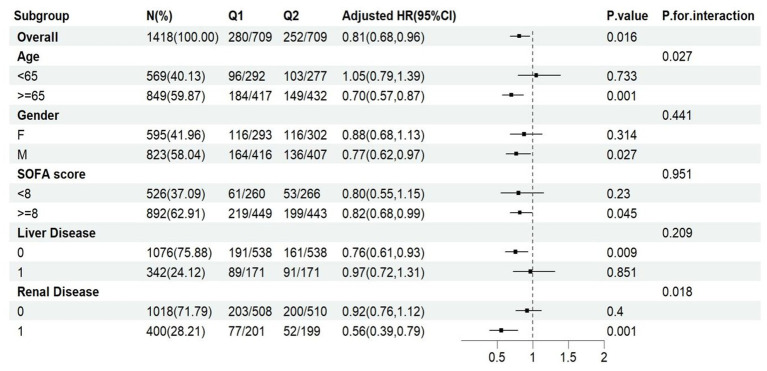
Subgroup analysis for association between ACR and 28-day all-cause mortality. Q1 (ACR ≤1846.154), Q2 (ACR >1846.154). HR, hazard ratio; CI, confidence interval.

Forest plots illustrated the relationship between ACR and 28-day all-cause mortality in sepsis patients with different clinical characteristics (Figure 4). A negative correlation was observed between ACR and 28-day all-cause mortality in patients aged over 65, male patients, patients with SOFA scores above 8, patients without liver disease, patients with renal disease (*p* < 0.05). The interaction test results indicated a significant interaction between age and ACR, as well as renal disease and ACR (*p* < 0.05). No significant interactions were observed in other subgroups (*p* > 0.05).

### 3.6 Assessment of predictive ability

**Figure 5:**
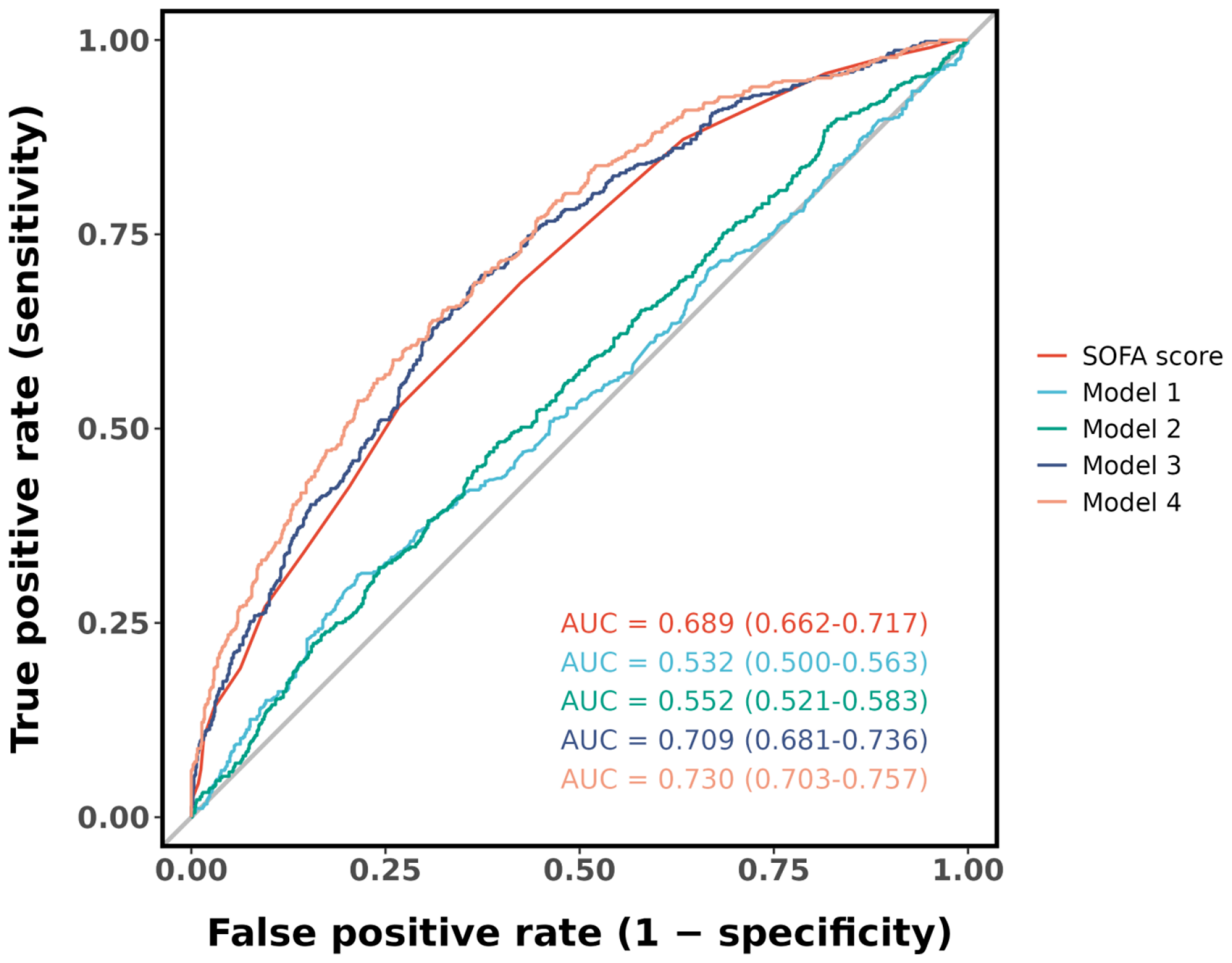
ROC curve for predicting 28-day all-cause mortality. Model 1: no covariates were adjusted. Model 2: adjusted for age, gender, and BMI. Model 3: adjusted for age, gender, BMI, and SOFA score. Model 4: adjusted for age, gender, BMI, SOFA score, white blood cell, hemoglobin, and lactate.

Evaluated the predictive ability of the SOFA score, Model 1 to Model 4 for determining 28-day all-cause mortality in sepsis, ROC curve analysis results were shown in Figure 5, with average AUC values of 0.689, 0.532, 0.552, 0.699, 0.709, and 0.730. The DeLong test showed that the AUC value of Model 4 was statistically significantly different from the AUC value of SOFA score and other models (*p* < 0.05), as shown in Supplementary Table 5.

## 4 Discussion

In this study, we analyzed the MIMIC-IV database to explore the association between ACR and the prognosis of sepsis. Although the Kaplan–Meier survival analysis and univariate COX regression did not show statistically significant differences, we observed a trend toward lower 28-day all-cause mortality in the high ACR group compared to the low ACR group. The multivariate COX regression analysis demonstrated that sepsis patients with a high ACR value might be associated with a low 28-day mortality risk. In addition, RCS analysis revealed a nonlinear association between increased ACR values and mortality risk. When ACR was below 2,300, it showed a negative association with mortality. However, no significant association was observed when ACR exceeded 2,300.

Subgroup analysis suggested that ACR was not consistently associated with 28-day all-cause mortality in sepsis patients with different clinical characteristics. Notably, ACR was associated with 28-day all-cause mortality in elderly patients (age ≥65), male patients, patients with high SOFA score (SOFA score ≥8), patients with renal disease and patients without liver disease. Interaction analysis revealed a significant interaction between age and ACR, as well as between renal disease and ACR. Older sepsis patients with lower ACR levels had a higher mortality, which interaction may be attributed to age-related renal function decline. Renal disease patients with a lower ACR level exhibited a higher mortality risk. Low ACR level may indicate high creatinine level. Elevated serum creatinine was associated with severe renal failure, which itself was significantly correlated with increased mortality risk in sepsis (15). This finding underscored the necessity of considering age and renal disease as effect modifiers when evaluating the prognostic value of ACR in sepsis patients.

The ROC curve analysis showed that the largest AUC value was achieved when combining ACR with age, gender, BMI, SOFA score, WBC, HGB, LAC. Compared with the SOFA score and other models, this combination may have better predictive performance for the 28-day all-cause mortality of sepsis patients.

Inflammatory response, oxidative stress, and endothelial dysfunction are involved in the occurrence and development of sepsis (16–18). In sepsis, decreased serum albumin level is observed due to factors such as inflammation, oxidative stress, and endothelial injury (19–21). The reduction of serum albumin is associated with poor outcomes in sepsis patients, which can serve as a predictor of mortality risk in sepsis (22). However, the optimal range of serum albumin levels in sepsis patients remains unknown. Acute kidney injury (AKI) is a common and serious complication of sepsis (23). Serum creatinine serves as a crucial indicator of renal function and plays a major role in predicting outcomes for sepsis patients. Numerous studies have demonstrated that fluctuations in serum creatinine level is strongly associated with both the onset and prognosis of AKI in sepsis (24, 25). Elevated serum creatinine typically indicates renal impairment, and septic patients with AKI face a substantially higher mortality risk (26, 27). Importantly, elevated creatinine level constitutes an independent risk factor for increased mortality in sepsis. One study examining the relationship between serum creatinine changes and mortality during the first 24 h of hospitalization found that a creatinine increase of ≥0.3 mg/dL had predictive value for sepsis-related deaths (28). Consequently, serum creatinine has emerged as a key prognostic marker in sepsis, and monitoring its dynamic changes may enable early identification of high-risk patients, allowing timely interventions to improve clinical outcomes.

Despite the importance of serum albumin as a prognostic indicator for sepsis, incorporating serum creatinine and examining the ACR value provide a methodologically superior approach in prognostic studies, as serum albumin level is often confounded by renal function. Previous studies on ACR had primarily focused on cardiovascular and cerebrovascular diseases. A single-center prospective cohort study reported that the lowest ACR tertile was significantly associated with a higher risk of heart failure-related mortality compared to the highest tertile (11). Similarly, a retrospective analysis revealed that low ACR levels independently predicted increased one-year all-cause mortality in ICU-admitted heart failure patients, with a threshold effect serving as an early warning marker for high-risk individuals (29). Additionally, ACR is inversely correlated with short-term clinical outcomes following PCI in ST-elevation myocardial infarction patients, with lower ACR corresponding to poorer prognosis (13). In cerebrovascular disease, a retrospective study demonstrated that admission ACR value strongly predicted in-hospital and long-term mortality, as well as stroke severity, in patients undergoing carotid artery stent implantation (14).

The present study explores the association of ACR with sepsis prognosis. Our findings align with prior research: higher ACR value is associated with reduced mortality risk in sepsis patients, especially when ACR value is less than 2,300. Nevertheless, the limitations of this study should be acknowledged. First, the diagnostic criteria for sepsis is variable, and the determination of ACR threshold is data-driven and lacks external validation, which may lead to some bias in sample selection. Secondly, although the propensity score matching controlled for confounding factors, it greatly reduced the sample size, potentially limiting the generalizability of the conclusions. Thirdly, the lack of validation in the ROC analysis limits the generalizability of our findings. We are committed to addressing this issue comprehensively, and our future research will focus on externally validating the ROC analysis. First, we will identify appropriate external datasets that are comparable to our study population in terms of key characteristics, such as disease type and patient demographics. These datasets may be sourced from public databases, collaborative research projects, or published literature. Subsequently, we will replicate our ROC analysis using these new datasets. Through this process, we aim to continuously optimize our model, refining it to enhance its clinical applicability. Finally, the present study only explored the relevance between the initially measured ACR and sepsis prognosis, overlooking the impact of dynamic changes in this ratio on outcomes. Future multi-center prospective clinical trials are needed to rigorously and comprehensively evaluate the role of ACR in sepsis prognosis, particularly the influence of its longitudinal trajectories.

## 5 Conclusion

A higher ACR value may associated with a lower 28-day all-cause mortality risk, especially when ACR value is less than 2,300. Moreover, ACR has some predictive power for adverse outcomes in sepsis patients. However, given the limitations of this study, these results require further validation in high-quality, multicenter, prospective clinical trials.

## Data Availability

The original contributions presented in the study are included in the article/Supplementary material, further inquiries can be directed to the corresponding author.

## Footnotes

^1^ https://postgres.ac.cn/

^2^ https://www.navicat.com.cn/

^3^ www.mstata.com
