# Supplementary figures and images for: Association between the serum albumin-to-creatinine ratio and 28-day all-cause mortality in sepsis: a retrospective cohort study
Source: Front Med (Lausanne). 2025 Sep 4;12:1540647. doi: 10.3389/fmed.2025.1540647 (PMC12443701; doi:10.3389/fmed.2025.1540647)

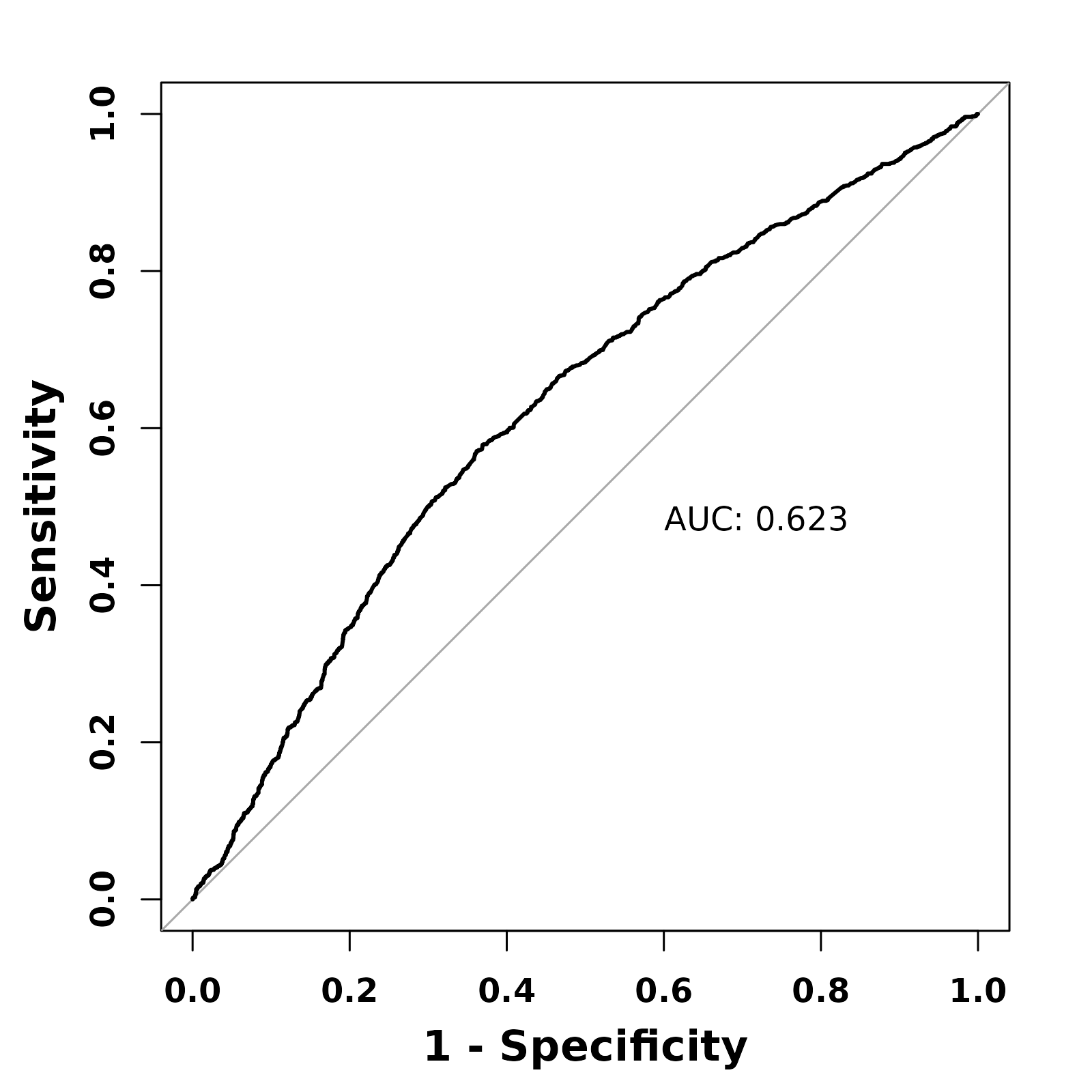

Supplement: Supplementary file 1 [file Image_1.jpeg]
