# Supplementary material for: Association between the serum albumin-to-creatinine ratio and 28-day all-cause mortality in sepsis: a retrospective cohort study
Source: Front Med (Lausanne). 2025 Sep 4;12:1540647. doi: 10.3389/fmed.2025.1540647 (PMC12443701; doi:10.3389/fmed.2025.1540647)
Supplement: Supplementary file 2 [file Table_1.docx]

**Supplementary Table 1 The Optimal Cut-off Value of ACR**

| AUC | Direction | Cut point | Metric Score: Youden | Sensitivity | Specificity | PPV | NPV | Accuracy | Precision | Recall | TP | FP | TN | FN |
| --- | --- | --- | --- | --- | --- | --- | --- | --- | --- | --- | --- | --- | --- | --- |
| 0.623 | <= | 1,846.154 | 0.2096071 | 57.9% | 63.1% | 44.8% | 74.3% | 61.3% | 44.8% | 57.9% | 801 | 986 | 1,685 | 583 |
| PPV positive predictive value；NPV negative predictive value；TP true positive；FP false positive；TN true negative；FN false negative. | | | | | | | | | | | | | | |
