# Supplementary material for: Association between the serum albumin-to-creatinine ratio and 28-day all-cause mortality in sepsis: a retrospective cohort study
Source: Front Med (Lausanne). 2025 Sep 4;12:1540647. doi: 10.3389/fmed.2025.1540647 (PMC12443701; doi:10.3389/fmed.2025.1540647)
Supplement: Supplementary file 3 [file Table_2.docx]

Supplementary Table2 Proportional Hazard Assumption Test

| **Cox Regression** | **Global Schoenfeld Test** | | |
| --- | --- | --- | --- |
|  | **Χ²** | **df** | **P value** |
| **Model 1** | 0.61 | 1 | 0.4337 |
| **Model 2** | 3.26 | 4 | 0.5154 |
| **Model 3** | 4.01 | 5 | 0.5484 |
| **Model 4** | 8.50 | 8 | 0.3864 |
