# Supplementary material for: Association between the serum albumin-to-creatinine ratio and 28-day all-cause mortality in sepsis: a retrospective cohort study
Source: Front Med (Lausanne). 2025 Sep 4;12:1540647. doi: 10.3389/fmed.2025.1540647 (PMC12443701; doi:10.3389/fmed.2025.1540647)
Supplement: Supplementary file 4 [file Table_3.docx]

**Supplementary Table 3 Variance Inflation Factor Analysis for Covariates**

| **Model** | **Variable** | **VIF** |
| --- | --- | --- |
| **Model 2** | Age(years) | 1.028 |
|  | Gender | 1.009 |
|  | BMI | 1.035 |
| **Model 3** | Age(years) | 1.103 |
|  | Gender | 1.010 |
|  | BMI | 1.029 |
|  | SOFA score | 1.088 |
| **Model 4** | Age(years) | 1.112 |
|  | Gender | 1.033 |
|  | BMI | 1.063 |
|  | SOFA score | 1.293 |
|  | White blood cell | 1.015 |
|  | Hemoglobin | 1.028 |
|  | Lactate | 1.221 |

BMI body mass index,SOFA sequential organ failure assessment.
