# Supplementary material for: Association between the serum albumin-to-creatinine ratio and 28-day all-cause mortality in sepsis: a retrospective cohort study
Source: Front Med (Lausanne). 2025 Sep 4;12:1540647. doi: 10.3389/fmed.2025.1540647 (PMC12443701; doi:10.3389/fmed.2025.1540647)
Supplement: Supplementary file 5 [file Table_4.docx]

Supplementary Table 4 Threshold Effect Analysis of ACR on Mortality

|  | **Adjusted HR (95% CI)*** | **P-value** |
| --- | --- | --- |
| **Fitting by standard linear regression model** | 1.0000 (0.9999 - 1.0001) | 0.378 |
| **Fitting by piecewise linear regression model (break-point = 2306.1)** |  |  |
| ACR < 2300 | 0.9997 (0.9995 - 0.9999) | 0.007 |
| ACR ≥ 2300 | 1.0001 (1.0000 - 1.0002) | 0.130 |
| **Log likelihood ratio** |  | 0.005 |
| *Adjusted for: age, gender, BMI, sofa score, white blood cell, hemoglobin, lactate | | |
