# Supplementary material for: Association between the serum albumin-to-creatinine ratio and 28-day all-cause mortality in sepsis: a retrospective cohort study
Source: Front Med (Lausanne). 2025 Sep 4;12:1540647. doi: 10.3389/fmed.2025.1540647 (PMC12443701; doi:10.3389/fmed.2025.1540647)
Supplement: Supplementary file 6 [file Table_5.docx]

**Supplementary Table5 The DeLong Test of AUC**

| Model | AUC Difference | Z Value | P Value |
| --- | --- | --- | --- |
| Model 1 vs Model 2 | 0.020 (0.532 ~ 0.552) | -1.03 | 0.303 |
| Model 1 vs Model 3 | 0.177 (0.532 ~ 0.709) | -8.55 | <0.001 |
| Model 1 vs Model 4 | 0.198 (0.532 ~ 0.730) | -9.64 | <0.001 |
| Model 1 vs SOFA | 0.157 (0.532 ~ 0.689) | -7.42 | <0.001 |
| Model 2 vs Model 3 | 0.157 (0.552 ~ 0.709) | -8.46 | <0.001 |
| Model 2 vs Model 4 | 0.178 (0.552 ~ 0.730) | -9.57 | <0.001 |
| Model 2 vs SOFA | 0.137 (0.552 ~ 0.689) | -5.89 | <0.001 |
| Model 3 vs Model 4 | 0.021 (0.709 ~ 0.730) | -3.44 | <0.001 |
| Model 3 vs SOFA | 0.019 (0.709 ~ 0.689) | 2.66 | 0.008 |
| Model 4 vs SOFA | 0.040 (0.730 ~ 0.689) | 4.35 | <0.001 |

Model 1 : no covariates were adjusted
Model 2 : adjusted for age, gender, and BMI
Model 3 : adjusted for age, gender, BMI, and sofa score
Model 4 : adjusted for age, gender, BMI, sofa score, white blood cell, hemoglobin, and lactate
